# Optimizing low-protein diets with edible dock powder: Integrated effects on growth performance, slaughter quality, Organ weights, Muscle quality, and Cecal microbiota in growing Sanhua geese

**DOI:** 10.1016/j.psj.2024.104476

**Published:** 2024-10-31

**Authors:** Xianze Wang, Huiying Wang, Yi Liu, Guangquan Li, Yunzhou Yang, Cui Wang, Shaoming Gong, Daqian He, Shufang Chen, Huiyan Jia

**Affiliations:** a Institute of Animal Husbandry and Veterinary Science, Shanghai Academy of Agricultural Science, Shanghai, 201106, PR China; b Institute of Livestock and Poultry, Ningbo Academy of Agricultural Sciences, Ningbo 315000, PR China

**Keywords:** Native Chinese goose, Dietary protein reduction, Rumex K-1, Growth efficiency, Gut microbiota

## Abstract

This study evaluated the effects of supplementing low-protein diets with Edible Dock Powder (**EDP**) on the growth performance, slaughter traits, serum biochemical parameters, muscle quality, and cecal microbiota of Sanhua geese. A total of 288 healthy, five-week-old Sanhua geese were randomly assigned to six dietary treatments in a 3 × 2 factorial design, with three crude protein levels (16.00 %, 14.50 %, and 13.00 %) and two levels of EDP supplementation (0 % and 2.50 %). Two-way ANOVA and Duncan's multiple range test were used for statistical analysis. EDP supplementation significantly increased average daily gain (**ADG**) and improved feed-to-gain ratio (**F/G**) during both growth phases (P<0.01). Lower protein levels significantly reduced average daily feed intake (**ADFI**) and increased the apparent digestibility of gross energy (**ADGE**) (P<0.01). EDP significantly improved slaughter rate and eviscerated yield (P<0.05), while reducing liver weight and webbed feet yield (P<0.01). Reduced protein levels decreased serum globulin (**GLB**) and increased blood urea nitrogen (**BUN**) levels (P<0.05), with significant interactions between protein levels and EDP supplementation (P<0.05). EDP also significantly altered the cecal microbiota composition, reducing the relative abundance of *Actinobacteria, Megamonas*, and *Collinsella* (P<0.05), and affecting KEGG pathways related to protein modification and secondary metabolite degradation (P<0.05). In conclusion, EDP supplementation in low-protein diets improved growth performance, slaughter characteristics, and cecal microbiota, showing potential as a sustainable feed additive for reducing environmental impact and improving the economic efficiency of poultry production.

## Introduction

Amidst the prevailing global economic and environmental pressures, the livestock industry faces sustained challenges concerning the stable supply of food and feed resources. Influenced by multiple factors such as climate change, land degradation, and biodiversity loss, these resources are under strain, escalating the demand for more sustainable and economically efficient feed resources (Govoni et al., 2023; Rauw et al., 2020). In this context, the development and utilization of feed resources that can reduce environmental burdens while enhancing production efficiency have become increasingly vital. Low-protein diets, as a resource-saving strategy with potential environmental advantages, have garnered significant attention (Mahmood et al., 2024;Liu et al., 2023; Van et al., 2023). By reducing the protein content in diets, they contribute to lowering feed costs and reducing nitrogen emissions, thereby mitigating environmental pressure (Kim et al., 2020; Attia et al 2020). However, these diets may negatively impact animal growth performance and health, as evidenced by reduced growth rates and lower feed conversion efficiency (Chrystal et al., 2020; Hounkpêvi et al., 2024). Thus, the search for feed additives that can improve the nutritional effectiveness of low-protein diets is essential.

Edible Dock (Rumex patientia L. × Rumex tianschanicus cv. Rumex K-1), a hybrid developed through the crossbreeding of Rumex patientia L. and Rumex tianschanicus cv., represents an efficient feed resource (Liu et al., 2024). Its fresh leaves have a high crude protein content of 35.8 g/100 g, comparable to soy protein, and are rich in amino acids, accounting for about 22 % of its composition. Additionally, it contains an abundance of vitamin C, minerals, and various bioactive compounds, demonstrating potential value as a feed additive (Cao et al., 2024). The plant's high ecological plasticity and adaptability facilitate its application under diverse feeding conditions (Li et al., 2023).

Geese occupy an important position in the global poultry industry, especially in countries like China, Poland, and Germany, where goose meat is favored for its unique flavor and nutritional value. The Sanhuang (Tri-colored) goose, a native breed of China, is recognized for its distinct three-colored plumage: primarily white, with a typically black head and gray or brown patches on the body. This breed is notable for its large size, with adult geese typically weighing between 5 to 6 kilograms, and is extensively farmed across China due to its rapid growth rate and tolerance to roughage (Yan et al., 2019; Xi et al., 2020). The strong disease resistance and adaptability of the Sanhuang goose also make it a frequent subject of waterfowl research.

Despite the high nutritional value, high yield, and strong adaptability of Edible Dock, its application in poultry, particularly in meat geese production, remains limited. This study aimed to investigate the effects of EDP supplementation in low-protein diets on the growth performance, apparent digestibility, slaughter characteristics, serum biochemical parameters, muscle quality, and cecal microbiota of Sanhua geese. Through systematic experimental design and scientific data analysis, we seek to validate whether EDP can effectively alleviate the nutritional limitations of low-protein diets, thereby enhancing the growth performance of Sanhuang geese, maintaining slaughtering traits, and improving gut health. This research could provide an economically viable feed additive solution and potentially offer a new sustainable strategy for the livestock industry.

## Research methods and materials

The animal study protocol was approved by the Animal Care and Use Committee of the Shanghai Academy of Agricultural Sciences (SAASPZ0522050) and implemented in accordance with the 'Experimental Animal—Guidelines for Welfare' (GB/T 42011-2022) and the Experimental Animal Care and Use Guidelines of China (EACUGC2018-01). Every effort was made to minimize the suffering of the geese involved in this study.

### Geese and housing

In this study, we selected 288 healthy five-week-old Sanhua geese for experimentation. These geese were housed in a goose shed containing several enclosures, each measuring 2 meters in length, 2 meters in width, and 0.8 meters in height. Each enclosure was equipped with sufficient feeders and water troughs, allowing the geese free access to food and water. The facility was equipped with an advanced temperature and humidity control system, ensuring that the environmental temperature was maintained at 16 ∼ 22°C and the humidity level at 50 % ∼ 65 %. This setup aimed to provide an ideal growth environment for the geese. This six-week experiment was conducted at the Zhuanghang Comprehensive Experimental Station of the Shanghai Academy of Agricultural Sciences. Throughout the experimental period, stringent immunization and disease prevention protocols of the experimental station were followed to ensure the accuracy of the experiment and the welfare of the animals.

### Experimental setup and treatment administration

In this investigation, 288 Sanhuang geese were sourced from the Xiangtian Ge Family Farm in Hexian, Ma'anshan City, Anhui Province. The Edible Dock Powder (EDP) was supplied by Shaanxi Sinuote Biotechnology Co., Ltd., located in Xi'an, Shanxi Province. Prior to initiating the experiment, each goose was weighed and randomly assigned to one of six treatment groups, each comprising six replicates with eight geese per replicate equally divided between males and females. The dietary treatments were structured as follows: Group A was administered a diet containing 16.00 % crude protein (CP); Group B consumed a diet with 14.50 % CP; Group C was provided a diet with 13.00 % CP; Group D received a diet containing 16.00 % CP supplemented with 2.50 % EDP; Group E was given a diet with 14.50 % CP supplemented with 2.50 % EDP; and Group F consumed a diet with 13.00 % CP and supplemented with 2.50 % EDP. The diet formulations for each group were carefully designed based on the nutritional standards for commercial meat geese as recommended by DB37/T 2784-2016, to meet the nutritional requirements of modern meat geese and accurately reflect the nutritional characteristics and inclusion levels of EDP.

### Nutrient composition detection

Crude protein (CP) analysis was performed following the protocols established by the Association of Official Analytical Chemists (AOAC, 2005). Metabolizable energy (ME) was quantified using the methodology described by Miller and Judd (1984). Analysis of crude fiber (CF) adhered to the procedures specified by AOAC (2000), and the assessment of ether extract (EE) was conducted in accordance with AOAC (1930) guidelines. Calcium (Ca) content was analyzed using the procedures outlined in AOAC (1985a), while phosphorus (P) levels were determined following AOAC (1985b) methodologies. The amino acid profile of the Edible Dock Powder (EDP) was evaluated by Engel Testing Services (Shanghai) Co., Ltd., using the method prescribed by the Chinese national standard (GB/T 18246). The detailed nutritional composition of the feeds for each treatment group, including EDP, is comprehensively documented in Table 1, Table 2.

**Table 1:** Ingredients and nutrient compositions of experimental diets (air dry basis).

Ingredient, %	Diet treatment					
	A 16.00 % CP	B 14.50 % CP	C 13.00 % CP	D 16.00 % CP+*EDP*	E 14.50 % CP+*EP*	F 13.00 % CP+*EDP*
Corn						
Soybean (43 % protein)	24.29	19.69	15.61	21.73	18.09	13.82
Husks	4.53	4.79	5.52	3.14	4.35	4.64
Edible Dock Powder (EDP)	0.00	0.00	0.00	2.50	2.50	2.50
Sprouting corn bran	0.00	0.98	0.72	2.50	0.79	1.23
Limestone	0.00	0.06	0.32	0.29	0.44	0.99
Dicalcium Phosphate	1.05	1.05	1.07	0.95	1.00	1.02
Premix^2^	3.00	3.00	3.00	3.00	3.00	3.00
Soybean oil	3.00	2.83	2.81	2.54	2.41	2.47
Salt	0.10	0.10	0.10	0.10	0.10	0.10
Nutritional leve1^1^						
Apparent Metabolizable Energy, MJ/kg	11.80	11.78	12.15	11.92	12.10	12.11
Crude Protein, %	16.03	14.61	13.23	15.72	14.53	13.32
Crude Fibre, %	4.00	4.02	4.10	4.07	4.19	4.17
Ether Extract, %	5.55	5.49	5.53	5.11	5.05	5.17
Lysine, %	0.92	0.79	0.68	0.88	0.77	0.66
Methionine + Cystine, %	0.63	0.58	0.52	0.61	0.56	0.51
Arginine, %	1.09	0.95	0.82	1.02	0.92	0.78
Tryptophan, %	0.20	0.18	0.15	0.20	0.18	0.16
Threonine, %	0.71	0.65	0.57	0.73	0.64	0.57
Valine, %	0.65	0.56	0.47	0.63	0.55	0.46
Calcium, %	1.00	1.00	1.00	1.00	1.00	1.00
Non-phytate Phosphorus, %	0.40	0.40	0.40	0.40	0.40	0.40

1 The apparent metabolizable energy (AME) and crude protein (CP) values are measured. Other nutrient composition values are calculated based on standard estimation method

2 The premix provides per kilogram of feed: Vitamin A 8,500 IU, Vitamin B1 2.2 mg, Vitamin B2 4 mg, Pantothenic acid 10 mg, Vitamin B6 3 mg, Vitamin B12 20 mg, Vitamin D3 1,600 IU, Vitamin E 20 IU, Vitamin K3 2 mg, Biotin 0.1 mg, Folic acid 0.4 mg, Niacin 60 mg, Choline 1,400 mg, Copper 6 mg, Iron 80 mg, Manganese 100 mg, Zinc 80 mg, Iodine 0.42 mg, Selenium 0.3 mg, Calcium 3 g, Phosphorus 0.99 g.

**Table 2:** Ingredient composition of *EDP*.

Nutritional level^1^	Nutrients
Crude Protein, %	30.00
Metabolizable Energy, MJ / kg	14.60
Ca, %	1.08
P, %	0.45
Ether Extract, %	0.50
Crude Fibre, %	17.20
Lysine, %	1.22
Methionine + Cystine, %	0.52
Arginine, %	1.03
Tryptophan, %	0.46
Threonine, %	1.08
Valine, %	1.29

1 The components of *MLP* are analytical values.

### Growth performance measurement

At the beginning (5 weeks old) and end (11 weeks old) of the study, each Sanhua goose was weighed after an 8-hour fast to determine its initial body weight (IBW) and final body weight (FBW). These measurements were used to calculate the average daily weight gain (ADG) for each goose. Throughout the study, we meticulously recorded the feed consumption of each treatment group to compute the average daily feed intake (ADFI) and feed-to-gain ratio (F/G).

The mathematical formulas used to evaluate growth performance are as follows:

ADG = (FBW - IBW) / Number of Days in the Experiment
F/G = Total Feed Consumption / Total Weight Gain
ADFI = F/G × ADG

### Determination of apparent digestibility of feed gross energy and crude protein

In the final week of the experiment (11 weeks of age), one goose was randomly selected from each replicate within every treatment group, resulting in six geese per group, and placed in metabolic cages for feces and feed sample collection. The feces collection period lasted for five consecutive days. During the collection period, all feces were gathered, and feathers and debris were removed. The samples were then treated with 10 % hydrochloric acid and stored in iron boxes at -20°C. The feces samples were dried in an oven at 65°C to a constant weight, ground to pass through a 40-mesh sieve, transferred into sample bags, and labeled. To evaluate the acid-insoluble ash (AIA) content in the feed and feces, appropriate amounts of each sample were taken and processed according to the AOAC (1942) method. Using AIA in the feed and feces as an indicator, the apparent digestibility of crude protein and gross energy in the feed was calculated using the following formulas:

$$\text{ADGE}\%=\left(1-\frac{\text{AI}A_{\text{feed}}\times GE_{\text{feces}}}{\text{AI}A_{\text{feces}}\times GE_{\text{feed}}}\right)\;\times \;100\%$$

$$\text{ADCP}\%=\left(1-\frac{\text{AI}A_{\text{feed}}\times CP_{\text{feces}}}{\text{AI}A_{\text{feces}}\times CP_{\text{feed}}}\right)\;\times \;100\%$$

ACPD: Apparent Digestibility of Crude Protein
ADGE: Apparent Digestibility of Feed Gross Energy
AIA_feed_: Acid-Insoluble Ash in Feed
AIA_feces_: Acid-Insoluble Ash in Feces
CP_feed_: Crude Protein Content in Feed
CP_feces_: Crude Protein Content in Feces
GE_feed_: Gross Energy Content in Feed
GE_feces_: Gross Energy Content in Feces

### Carcass performance and relative organ weight measurement

The measurement of carcass traits was conducted according to the method described by Yu et al. (2022). Specifically, after slaughter, the feathers of the geese were carefully removed, and the carcass weight was recorded. The birds were then eviscerated, with the trachea, esophagus, intestines, spleen, pancreas, gallbladder, reproductive organs, contents of the gizzard, and the gizzard lining removed. The semi-eviscerated carcass weight was measured and recorded. Subsequently, the heart, liver, spleen, gizzard, and webbed feet were carefully separated and weighed. After removing these organs and abdominal fat, the eviscerated carcass weight was directly measured and recorded. The carcass weight, semi-eviscerated carcass weight, and eviscerated carcass weight were all expressed as percentages of the live body weight. The calculation of relative organ weight is performed by taking the ratio of the specific organ's weight to the live body weight before slaughter, with results expressed in g/kg (NY/T 2793-2015).Additionally, the entire intestinal tract was rinsed with physiological saline to remove the contents, and the intestinal weight was measured. Finally, a caliper was used to measure morphological parameters of the carcass, including breast width, breast depth, and keel length. Formulas for calculating carcass traits are as follows:

Carcass weight yield = (Carcass Weight / Live body weight) × 100 %
Semi-eviscerated carcass yield = (Semi-eviscerated carcass weight / Live body weight) × 100 %
Eviscerated carcass yield = (Eviscerated carcass weight / Live body weight) × 100 %
Webbed feet yield= (Webbed feet weight / Live body weight) × 100 %
The formula for calculating the relative organ weight is:
Relative organ weight = Organ weight / live body weight

### Breast muscle quality measurement

After weighing, the right pectoral muscle was carefully removed and stored in a refrigerator at 4°C. Within 45 minutes postmortem, the pH value of the muscle was measured using a pH meter (S2-Food, Mettler-Toledo, Switzerland), with three measurements taken for accuracy. The brightness (L*), redness (a*), and yellowness (b*) of the muscle were evaluated using a colorimeter (OPTO-STAR, MATTHAUS, Germany), with three repeated measurements, and the average values were used for statistical analysis.

### Serum biochemical parameters measurement

Prior to slaughter, 5 mL of blood was carefully drawn from the wing vein to isolate serum for biochemical analyses. The serum samples were subsequently forwarded to Shanghai Renjie Biotechnology Co., Ltd. for analytical processing. The biochemical indices evaluated included total protein (TP), albumin (ALB), globulin (GLB), total cholesterol (TC), blood urea nitrogen (BUN), high-density lipoprotein cholesterol (HDL-C), low-density lipoprotein cholesterol (LDL-C), secretory immunoglobulin A (SIgA), and secretory immunoglobulin M (SIgM), providing a comprehensive profile of the metabolic and immune status of the animals.

### Cecum content 16S rRNA sequencing

During the slaughtering process, contents from the ceca of each goose were collected. The collected samples were immediately placed in cryovials and stored in liquid nitrogen before being sent to Shanghai Paisenno Biotechnology Co., Ltd. for DNA extraction. After assessing the quality and concentration, DNA was amplified via PCR using specific primers targeting the V3-V4 regions of the bacterial 16S rRNA gene (F: ACTCCTACGGGAGGCAGCA; R: GGACTACHVGGGTWTCTAAT). The amplified 16S rRNA gene fragments were then size-selected and purified through gel electrophoresis. The purified PCR products were used to construct sequencing libraries, which, after quality control, were sequenced on the Illumina MiSeq platform using a paired-end strategy. The raw sequencing reads were processed using the DADA2 tool for quality control, denoising, merging, and chimera removal to obtain high-quality ASVs. These ASVs were then annotated with species classification against the Greengenes database and used to construct a phylogenetic tree.

## Statistic and analysis

All data were initially processed using Microsoft Excel 2013, and subsequent analyses were conducted using SPSS 17.5, employing a general linear model for Two-Way ANOVA. When significant main effects or interactions were detected, post-hoc comparisons were performed using Duncan's multiple range test. For taxonomic classification from phylum to genus levels, we utilized the classify-sklearn algorithm in QIIME2, employing a pre-trained Naive Bayes classifier. Alpha diversity indices were calculated using the ggplot2 package within QIIME2. Additionally, non-metric multidimensional scaling (NMDS) was conducted using the Bray-Curtis distance matrix with the vegan package in R to illustrate the compositional differences in microbial communities via a two-dimensional ordination plot. Data are presented as mean ± standard error of the mean (SEM). Differences were considered significant at p < 0.05 and highly significant at p < 0.01.

## Result

### Growth performance

From 5 to 8 weeks of age, the ADFI of group A was significantly higher than that of the other groups (P<0.05), and its F/G was significantly higher than in groups B, E, and F. There were no significant differences in growth performance between the other groups (P>0.05). Lowering dietary protein levels and supplementing with EDP significantly reduced ADFI and F/G (P<0.01), but the interaction between these two factors was not significant (P>0.05). From 8 to 11 weeks of age, the ADG of groups D and F was significantly higher than that of groups A, B, and C (P<0.05), and group E's ADG was also significantly higher than that of group A (P<0.05). Additionally, the F/G of groups F and D was significantly lower than that of groups A and C (P<0.05). There were no significant differences in growth performance among groups A, B, and C (P>0.05), and no significant differences between groups D, E, and F (P>0.05). Supplementing with EDP significantly increased ADG and decreased F/G (P<0.01), while dietary protein levels had no significant effect on growth performance (P>0.05). The interaction between dietary protein levels and EDP was also not significant (P>0.05). During the entire 5 to 11-week period, the ADG of group F was significantly higher than that of groups A, B, and C (P<0.05), and its F/G was significantly lower than in groups A and C (P<0.05). Additionally, group F's final body weight (FBW) was significantly higher than that of group A (P<0.05). There were no significant differences in growth performance between the other groups during the 5-11 week period (P>0.05). Supplementing with EDP significantly improved ADG, reduced F/G, and increased FBW throughout the study (P<0.01). Dietary protein levels had no significant effect on growth performance, and the interaction between protein levels and EDP supplementation was not significant (P>0.05) (Table 3).

**Table 3:** Effects of low-protein diet supplemented with *EDP* on growth performance of Sanhua geese in growing period.

Items		5 to 8 weeks of age				8 to 11 weeks of age			5 to 11 weeks of age			
		Initial body weights, g	Average daily weight gain, g	Average daily feed intake, g	Feed-to-gain ratio	Average daily weight gain, g	Average daily feed intake, g	Feed-to-gain ratio	Average daily weight gain, g	Average daily feed intake, g	Feed-to-gain ratio	Final body weight
A	Groups	2065.74±52.11	70.83±1.57	194.79±4.41^A^	2.77±0.07^A^	25.10±1.36^C^	232.86±11.89	9.58±0.56^A^	47.43±1.17^B^	209.26±4.90	4.45±0.16^A^	4058.00±50.15^b^
B		2077.45±47.82	69.51±1.95	175.32±4.83^B^	2.52±0.04^B^	26.67±1.08^BC^	239.19±9.23	9.06±0.38^AB^	48.09±1.18^B^	207.26±5.05	4.31±0.07^AB^	4097.22±56.30^ab^
C		2093.40±37.46	70.27±2.05	179.95±5.22^AB^	2.57±0.05^AB^	26.93±1.44^BC^	255.30±12.89	9.61±0.47^A^	48.60±1.31^B^	217.64±5.68	4.49±0.10^A^	4134.62±62.18^ab^
D		2097.38±40.39	69.09±1.24	175.66±2.98^B^	2.54±0.05^AB^	33.56±1.34^A^	259.41±9.43	7.84±0.46^BC^	51.33±0.97^AB^	217.43±3.83	4.25±0.11^AB^	4253.17±49.27^ab^
E		2030.25±45.22	72.72±1.59	175.30±3.59^B^	2.42±0.06^B^	30.73±1.31^AB^	253.73±10.57	8.29±0.21^ABC^	51.73±1.11^AB^	214.77±4.43	4.16±0.10^AB^	4202.73±59.44^ab^
F		2020.90±47.78	74.74±1.76	174.47±4.07^B^	2.34±0.05^B^	34.46±1.27^A^	246.66±8.78	7.18±0.20^C^	54.60±1.17^A^	210.52±4.32	3.86±0.08^B^	4314.04±56.62^b^
16.00 %	level	2081.56±31.81	69.96±1.19	185.23±2.96^a^	2.66±0.04^A^	29.33±0.91	246.14±7.39	8.71±0.29	49.38±0.80	213.35±3.30	4.35±0.08	4155.58±36.63
14.50 %		2053.85±32.57	71.12±1.22	175.31±3.03^b^	2.47±0.04^B^	28.70±0.92	246.46±7.50	8.67±0.31	49.91±0.82	211.01±3.37	4.24±0.08	4149.98±39.55
13.00 %		2057.15±32.91	72.50±1.23	177.21±3.06^b^	2.45±0.04^B^	30.70±0.92	250.98±7.58	8.40±0.31	51.60±0.83	214.08±3.41	4.18±0.08	4224.33±39.96
0.00 %	Ratio	2078.87±26.23	70.20±0.98	183.36±2.44^b^	2.62±0.03^A^	26.23±0.75^B^	242.45±6.08	9.42±0.24^A^	48.04±0.66^B^	211.39±2.72	4.42±0.06^A^	4096.61±31.86^B^
2.50 %		2049.51±26.73	72.18±1.00	175.14±2.49^a^	2.44±0.03^B^	32.92±0.76^A^	253.27±6.16	7.77±0.26^B^	52.55±0.68^A^	214.24±2.77	4.09±0.07^B^	4256.65±32.46^A^
Level	*P*-value	0.801	0.333	0.046	0.001	0.300	0.879	0.730	0.139	0.799	0.286	0.338
Ratio		0.434	0.158	0.019	*P*<0.001	*P*<0.001	0.212	*P*<0.001	*P*<0.001	0.463	0.001	0.001
Level * Ratio		0.492	0.157	0.068	0.438	0.205	0.243	0.185	0.541	0.198	0.087	0.691

In the table, 'Ratio' refers to the proportion of *EDP* supplemented to the feed, with divisions at 0.00 % and 2.50 %. 'Level' denotes the crude protein level in the feed, categorized into 16.00 %, 14.50 %, and 13.00 %. 'Ratio × Level' indicates the interaction between the supplementation ratio of *EDP* and the crude protein level of the feed. Within the same column, identical or absence of uppercase or lowercase letters signifies no significant difference (*P* > 0.05), different lowercase letters denote significant differences *(P* < 0.05), and different uppercase letters indicate highly significant differences (*P* < 0.01). The same notation applies to the following tables."

### Apparent digestibility of feed gross energy and crude protein

The results of the ADCP and ADGE for geese in different treatment groups are shown in Table 4. There were no significant differences in ADCP values among the treatment groups (P>0.05). Dietary protein levels and EDP supplementation had no significant effect on ADCP, and the interaction between the two was also not significant (P>0.05). The ADGE values of groups C, D, E, and F were significantly higher than those of groups A and B (P<0.01). The ADGE of the 13.00 % protein level group was significantly higher than that of the 16.00 % and 14.50 % protein level groups (P<0.01). Additionally, the ADGE of the EDP-supplemented groups was significantly higher than that of the non-EDP groups (P<0.01). There was a significant interaction between dietary protein levels and EDP supplementation on ADGE (P<0.01)

**Table 4:** Effects of low-protein diet supplemented with *EDP* on apparent crude protein digestibility and apparent digestibility of feed gross energy in Sanhua geese.

Items		ADCP, %	ADGE, %
Groups	A	35.94±1.10	79.57±0.11^B^
	B	39.89±1.49	79.79±0.18^B^
	C	39.27±1.83	81.44±0.27^A^
	D	40.32±1.62	80.77±0.22^A^
	E	41.04±1.74	81.32±0.20^A^
	F	41.06±1.92	81.53±0.22^A^
Level	16.00 %	38.13±1.15	80.17±0.15^B^
	14.50 %	40.46±1.16	80.55±0.15^B^
	13.00 %	40.16±1.15	81.48±0.14^A^
Ratio	0.00 %	38.36±0.95	80.26±0.12^B^
	2.50 %	40.80±0.95	81.21±0.12^A^
P-value	Level	0.314	*P*<0.001
	Ratio	0.078	*P*<0.001
	Level * Dose	0.585	0.003

ACPD: Apparent Digestibility of Crude Protein

ADGE: Apparent Digestibility of Feed Gross Energy

### Carcass performance and carcass traits

The carcass traits are shown in Table 5. As shown, the carcass yield and eviscerated carcass yield of groups D and E were significantly higher than those of group A (P<0.05), with no significant differences observed among the other groups (P>0.05). The semi-eviscerated carcass yield showed no significant differences among the groups (P>0.05). In terms of breast dimensions, the breast width of groups D and E was significantly greater than that of groups A and B (P<0.01), with no significant differences among the other groups (P>0.05). The breast depth of groups E and F was significantly lower than that of groups B and D (P<0.05), with no significant differences observed among the other groups (P>0.05). Additionally, no significant differences in keel length were found among the groups (P>0.05). The supplementation of EDP significantly increased carcass yield and eviscerated carcass yield (P<0.05), while dietary protein levels had no significant effect on the measured carcass traits (P>0.05). However, there were significant interactions between dietary protein levels and EDP supplementation on eviscerated carcass yield, breast width, and breast depth (P<0.05)

**Table 5:** Effects of low-protein diet supplemented with *EDP* on carcass traits of Sanhua geese in growing period.

Items		Carcass weight yield, %	Semi-eviscerated carcass yield, %	Eviscerated carcass yield, %	Keel length, cm	Breast width, mm	Breast depth, mm
							121.69±1.15^ab^
	B	84.31±0.63^ab^	70.39±1.68	72.45±0.40^AB^	16.77±0.26	150.89±3.54^BC^	125.28±2.61^a^
	C	84.20±0.67^ab^	79.28±0.72	72.72±0.77^AB^	16.95±0.18	159.56±3.55^AB^	121.92±2.29^ab^
	D	85.83±0.49^a^	81.07±0.52	74.59±0.56^A^	17.08±0.23	163.40±2.28^A^	125.05±1.41^a^
	E	85.61±1.29^a^	81.20±0.60	74.26±0.56^A^	17.47±0.26	161.54±3.38^A^	118.52±2.27^b^
	F	84.50±2.65^ab^	79.42±0.82	72.65±0.66^AB^	17.73±0.34	157.66±4.32^AB^	116.03±1.90^b^
Level	16.00 %	84.32±0.49	79.57±0.62	72.55±0.47	17.18±0.21	155.38±2.27	123.37±1.36
	14.50 %	84.96±0.51	80.80±0.64	73.36±0.49	17.12±0.21	156.21±2.36	121.90±1.41
	13.00 %	84.35±0.51	79.35±0.64	72.68±0.49	17.34±0.21	158.61±2.36	118.98±1.41
Ratio	0.00 %	83.77±0.41^b^	79.25±0.51	71.90±0.39^B^	17.00±0.17	152.60±1.88^B^	123.96±1.12
	2.50 %	85.31±0.42^a^	80.56±0.53	73.83±0.40^A^	17.43±0.18	160.87±1.93^A^	119.87±1.51
*P*-value	Level	0.607	0.244	0.463	0.745	0.601	0.091
	Ratio	0.013	0.083	0.002	0.086	0.004	0.064
	Level * Dose	0.168	0.255	0.017	0.206	0.031	0.025

### Relative organ weight

Table 6 presents the relative organ weights across different treatment groups. The results indicate that there were no significant differences in the relative weights of the heart, liver, spleen, gizzard, and intestines among the groups (P>0.05). However, the supplementation of EDP significantly reduced the relative weight of the liver in geese (P<0.01). Dietary protein levels had no significant effect on the relative organ weights of the geese (P>0.05), and there was no significant interaction between dietary protein levels and the supplementation of EDP on the relative organ weights (P>0.05).

**Table 6:** Effects of low-protein diet supplemented with *EDP* on the relative organ weight of Sanhua geese in growing period.

Items		Heart yield, g/kg	Liver yield, g/kg	Spleen yield, g/kg	Gizzard yield,g/kg	Intestine yield, g/kg
Groups	A	0.65±0.02	1.71±0.07	0.10±0.03	3.86±0.08	3.10±0.12
	B	0.66±0.03	1.68±0.09	0.10±0.05	3.70±0.20	3.38±0.24
	C	0.72±0.03	1.66±0.03	0.11±0.02	3.47±0.18	3.50±0.14
	D	0.72±0.03	1.51±0.03	0.10±0.03	3.42±0.22	3.28±0.11
	E	0.67±0.01	1.53±0.04	0.10±0.05	3.36±0.18	3.16±0.18
	F	0.67±0.03	1.53±0.06	0.09±0.02	3.68±0.15	3.54±0.22
Level	16.00 %	0.69±0.02	1.61±0.04	0.10±0.01	3.64.±0.12	3.19±0.12
	14.50 %	0.67±0.02	1.60±0.04	0.10±0.01	3.53±0.12	3.27±0.12
	13.00 %	0.69±0.02	1.59±0.04	0.10±0.01	3.57±0.12	3.52±0.12
Ratio	0.00 %	0.68±0.01	1.68±0.03^A^	0.10±0.01	3.68±0.10	3.33±0.10
	2.50 %	0.69±0.01	1.52±0.03^B^	0.10±0.01	3.48±0.10	3.33±0.10
P-value	Level	0.584	0.975	0.984	0.801	0.149
	Ratio	0.730	0.003	0.757	0.178	0.988
	Level * Dose	0.064	0.814	0.726	0.150	0.491

### Breast muscle quality

The results of breast muscle quality are shown in Table 7. The pH values of the breast muscle in groups C and E were significantly lower than those in group A (P<0.05), while there were no significant differences in pH among the other groups (P>0.05). Dietary protein levels and EDP supplementation had no significant effect on breast muscle quality, and there was no significant interaction between the two (P>0.05). There were also no significant differences in breast muscle color indicators (L, a, b*) among the groups (P>0.05). Neither dietary protein levels nor EDP supplementation had a significant effect on breast muscle color, and there was no significant interaction between the two (P>0.05).

**Table 7:** Effects of low-protein diet supplemented with *EDP* on the breast muscle quality t of Sanhua geese in growing period.

Items		PH	L*	a*	b*
Groups	A	6.21±0.06^a^	5.50±0.62	17.56±1.37	8.28±0.70
	B	5.93±0.20^ab^	5.97±0.76	16.11±2.06	7.72±1.14
	C	5.75±0.07^b^	10.33±2.54	18.33±0.60	8.56±0.72
	D	5.87±0.10^ab^	8.67±1.11	18.16±1.19	8.45±0.98
	E	5.74±0.02^b^	8.33±0.56	18.45±1.06	8.94±0.78
	F	5.84±0.04^ab^	8.42±1.05	17.44±1.44	9.61±1.21
Level	16.00 %	6.04±0.07	7.08±0.92	17.86±0.96	8.36±0.67
	14.50 %	5.83±0.07	7.15±0.92	17.28±0.96	8.33±0.67
	13.00 %	5.80±0.07	9.38±0.92	17.89±0.96	9.09±0.67
Ratio	0.00 %	5.96±0.06	7.27±0.75	17.33±0.79	8.19±0.55
	2.50 %	5.81±0.06	8.47±0.75	18.02±0.79	9.00±0.55
*P*-value	Level	0.055	0.149	0.881	0.668
	Ratio	0.083	0.264	0.542	0.299
	Level * Dose	0.119	0.125	0.504	0.838

### Serum biochemical parameters

The results of serum biochemical indicators are shown in Table 8. The serum GLB level in group D was significantly higher than that in group F (P<0.01), with no significant differences among the other groups. Serum GLB levels decreased significantly as dietary protein levels decreased (P<0.05). EDP supplementation had no significant effect on serum GLB, but there was a significant interaction between dietary protein levels and EDP supplementation (P<0.05). Serum BUN levels in groups B and F were significantly higher than in group A (P<0.05), while there were no significant differences among the other groups (P>0.05). Serum BUN levels increased significantly as dietary protein levels decreased (P<0.05). EDP supplementation had no significant effect on serum BUN, but the interaction between dietary protein levels and EDP supplementation was significant (P<0.05). Dietary protein levels and EDP supplementation had no significant effects on serum TP, ALB, GLB, TC, LDL-c, HDL-c, SIgA, and SIgM (P>0.05).

**Table 8:** Effects of low-protein diet supplemented with EDP on the blood biochemical of Sanhua geese in growing period.

Items		TP, g/L	ALB, g/L	GLB, g/L	TC, mmol/L	LDL-c, mmol/L	HDL-c, mmol/L	BUN, mmol/L	SIgA, μg/mL	SIgM, μg/mL
Groups	A	96.35±5.83	52.32±3.28	43.88±2.84^AB^	4.73±0.40	2.50±0.19	2.18±0.26	4.85±0.52^b^	17.44±1.27	19.56±1.83
	B	98.53±3.47	52.88±2.83	45.55±1.44^AB^	5.14±0.49	3.48±0.28	1.60±0.34	7.83±0.59^a^	12.54±2.09	17.73±1.53
	C	98.18±5.02	52.85±2.57	45.35±3.49^AB^	4.12±0.23	2.62±0.23	1.47±0.20	6.67±0.62^ab^	16.18±1.32	16.86±0.62
	D	105.37±1.73	51.07±0.87	54.20±1.20^A^	4.21±0.33	2.46±0.26	1.68±0.19	6.31±0.60^ab^	14.57±0.96	18.62±1.29
	E	97.45±4.73	51.48±2.67	45.98±3.19^AB^	4.30±0.34	2.63±0.26	1.63±0.20	5.91±0.60^ab^	13.25±0.72	17.24±1.38
	F	86.75±3.30	48.45±2.51	38.35±1.49^B^	4.55±0.28	2.99±0.31	1.54±0.10	7.45±0.52^a^	14.74±1.77	17.76±1.31
Level	16.00 %	100.86±3.00	51.69±1.81	49.04±1.74^a^	4.47±0.25	2.48±0.18	1.93±0.17	5.58±0.41^b^	16.00±1.01	19.09±0.97
	14.50 %	97.99±3.00	52.18±1.81	45.77±1.74^ab^	4.72±0.25	3.05±0.18	1.61±0.17	6.87±0.41^ab^	12.90±1.01	17.49±0.97
	13.00 %	92.47±3.00	50.65±1.81	41.85±1.74^b^	4.33±0.25	2.81±0.18	1.51±0.17	7.06±0.41^a^	15.46±1.01	17.31±0.97
Ratio	0.00 %	97.69±2.44	52.68±1.48	44.93±1.42	4.66±0.21	2.87±0.15	1.75±0.13	6.45±0.33	15.38±0.83	18.05±0.79
	2.50 %	96.52±2.44	50.33±1.48	46.18±1.42	4.35±0.21	2.69±0.15	1.62±0.13	6.56±0.33	14.19±0.83	17.88±0.79
*P*-value	Level	0.149	0.831	0.023	0.558	0.099	0.170	0.031	0.085	0.374
	Ratio	0.738	0.271	0.537	0.296	0.423	0.483	0.827	0.312	0.88
	Level * Dose	0.069	0.788	0.005	0.199	0.072	0.389	0.016	0.461	0.786

Notes: TP: Total protein, ALB: Albumin, GLB: Globulin, BUN: Blood urea nitrogen, GLU: Glucose, TC: Total cholesterol, HDL-c: High-density lipoprotein cholesterol, LDL-c: Low-density lipoprotein cholesterol, SIgA: Secretory immunoglobulin A, SIgM: Secretory immunoglobulin M

### Cecum content 16S rRNA sequencing

#### Venn diagrams based on amplicon sequence variants (ASVs)

**Fig. 1:**
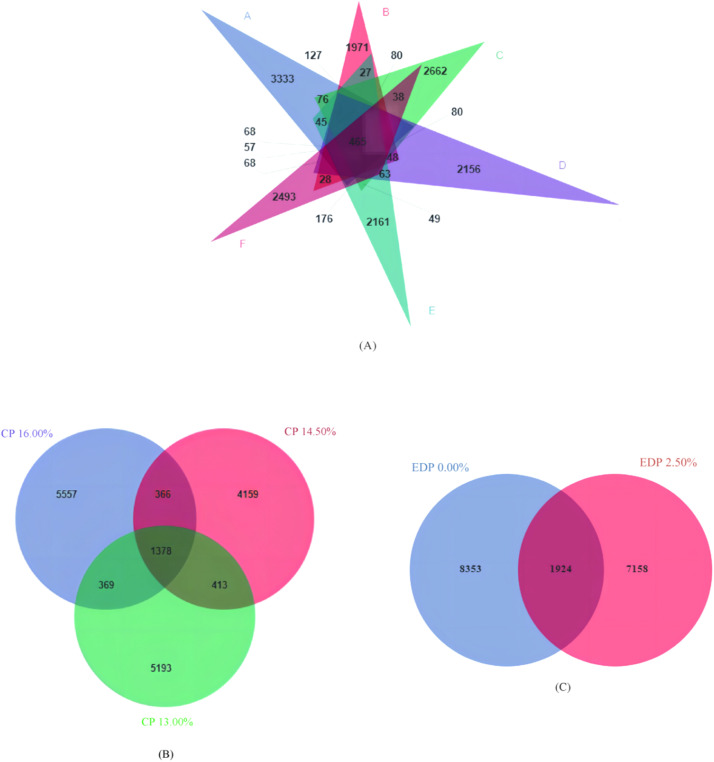
Venn diagram of ASV of cecum flora.

Fig. 1 presents the Venn diagrams illustrating the Amplicon Sequence Variants (ASVs) diversity within the cecal microbiota of geese subjected to different treatments. The number of ASVs shared across all treatment groups is 465. Specifically, group A possesses 3 333 unique ASVs, group B has 1 971 unique ASVs, group C has 2 662 unique ASVs, group D has 2 156 unique ASVs, group E has 2 161 unique ASVs, and group F has 2 493 unique ASVs as shown in Fig. 1 (A). With regards to dietary protein levels, there are 1 378 ASVs common across all levels, with unique ASVs numbering 5 557 for the 16 % CP protein level, 4 159 for the 14.5 % CP protein level, and 5 193 for the 13 % CP protein level as shown in Fig. 1 (B). As for different levels of EDP supplementation in the feed, there are 1 924 ASVs shared between the treatments, with 8 353 unique ASVs identified in the non-EDP supplemented group, and 7 158 unique ASVs in the EDP supplemented group as depicted in Fig. 1 (C)

#### Alpha diversity

**Fig. 2:**
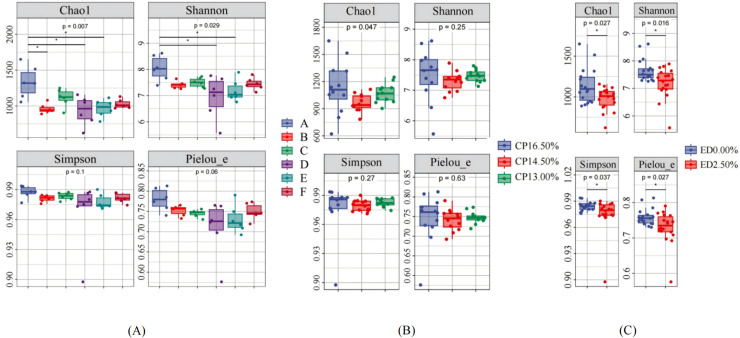
Effects of low protein diet supplemented with *EDP* on cecum flora.

The α-diversity of cecal contents is shown in Fig. 2. The Chao1 index of the cecal microbiota in group A was significantly higher than that in groups B, D, and E (P<0.05), and the Shannon index of group A was also significantly higher than that in groups D and E (P<0.05). There were no significant differences in the Chao1 and Shannon indices among the other groups (P>0.05). The Simpson index and Pielou_e index showed no significant differences among the treatment groups (P>0.05), as shown in Fig. 2 (A). Additionally, dietary protein levels had no significant effect on the α-diversity of the cecal microbiota (P>0.05), as shown in Fig. 2 (B). However, EDP supplementation significantly reduced the Chao1, Shannon, Simpson, and Pielou_e indices of the cecal microbiota (P<0.05), as shown in Fig. 2 (C)

Alpha diversity of Sanhua geese during growing period (*: P<0.05)

#### NMDS analysis of cecal microbiota

**Fig. 3:**
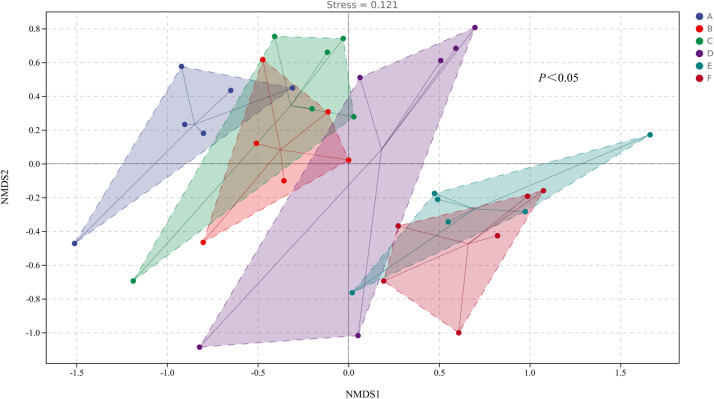
NMDS analysis of cecum flora.

Note: Based on the Bray-Curtis distance NMDS analysis, different colors and shapes represent samples from different group

Non-metric Multidimensional Scaling (NMDS) analysis was conducted on the cecal microbiota of various treatment groups to visualize the effects of different treatments on the composition of cecal microbiota (see Fig. 3). The analysis revealed clear clustering patterns, reflecting differences in microbial community structures among treatments, with a stress value of 0.121 indicating a good fit of the model to the data.

The NMDS plot demonstrated higher clustering between groups B and C, as well as E and F, indicating a higher similarity in microbial communities within these groups. Groups that did not receive EDP supplementation (A, B, C) tended to cluster together, showing similar microbial characteristics. In contrast, groups that received EDP supplementation (D, E, F) exhibited a distinctly different microbial community structure from those that did not receive EDP, highlighting the significant impact of EDP on cecal microbial diversity.

#### Taxonomic composition analysis at the phylum level in cecal microbiota

**Fig. 4:**
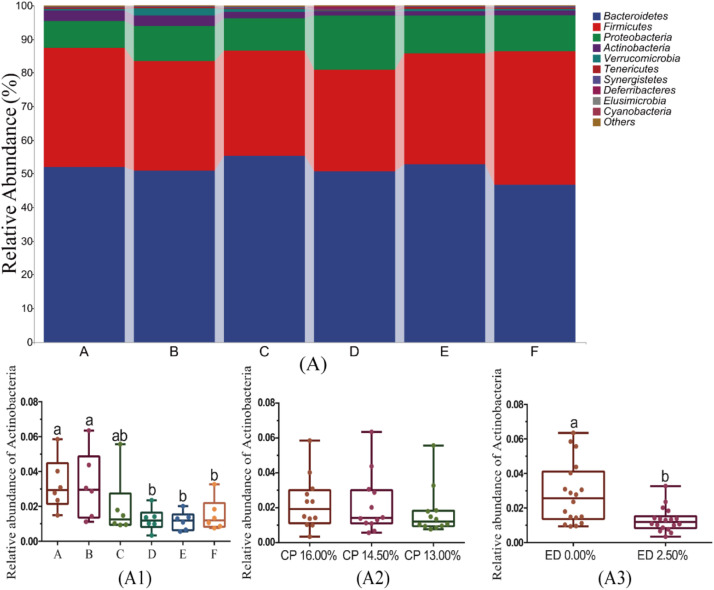
Taxonomic Composition and Abundance Analysis of Cecal Microbiota at the Phylum Level. Fig. 4A presents the taxonomic composition analysis of the top ten most abundant phyla in the cecal microbiota. Fig. 4A1 shows the abundance levels of phyla with significant differences across groups at the phylum level. Fig. 4A2 details the abundance levels of phyla with significant differences under different dietary protein levels, and Fig. 4A3 illustrates the abundance levels of these phyla under different levels of EDP supplementation in the feed.

Note: Different lowercase letters above the box plots indicate significant differences (P < 0.05).

Fig. 4 shows the composition of the top ten phyla in the cecal microbiota of geese. The microbial composition of all treatment groups was relatively similar, mainly including Bacteroidetes, Firmicutes, Proteobacteria, Actinobacteria, Verrucomicrobia, Tenericutes, Synergistetes, Deinococcus-Thermus, Elusimicrobia, and Cyanobacteria (Fig. 4A). The Actinobacteria abundance in groups A and B was significantly higher than in groups D, E, and F (P < 0.05, Fig. 4A1), while there were no significant differences in Actinobacteria abundance among the other groups (P>0.05). EDP supplementation significantly reduced the relative abundance of Actinobacteria in the cecal microbiota (P < 0.05, Fig. 4A3), while changes in dietary protein levels had no significant effect on Actinobacteria abundance (P>0.05, Fig. 4A2).

#### Genus-level composition analysis of cecal microbiota

**Fig. 5:**
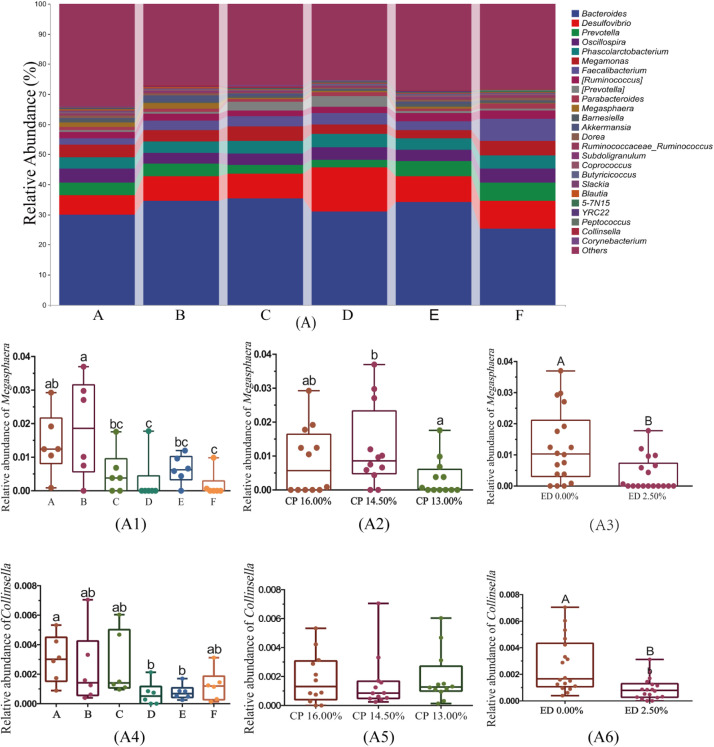
Taxonomic Composition and Abundance Analysis of Cecal Microbiota at the Genus Level. Fig. 5A presents the taxonomic composition analysis of the top 25 most abundant genera in the cecal microbiota. Fig. 5 details the abundance levels of genera with significant differences across groups at the genus level (A1, A4); the abundance levels of these genera under different dietary protein levels (A2, A5); and the abundance levels of these genera under different levels of EDP supplementation in the feed (A3, A6).

Note: Different lowercase letters above the box plots indicate significant differences (P < 0.05), while different uppercase letters indicate highly significant differences (P < 0.01).

Fig. 5A illustrates the composition of the top 25 genera in the cecal microbiota, highlighting significant differences in genera such as Megamonas and Collinsella among the treatment groups. The relative abundance of Megamonas in group A was significantly higher than in groups D and F (P<0.05), while in group B, it was significantly higher than in groups C, D, E, and F (P<0.05), with no significant differences among the other groups (P>0.05), as shown in Fig. 5A1. Both dietary protein levels and EDP supplementation significantly influenced Megamonas; its relative abundance initially increased and then significantly decreased with changes in protein levels (P<0.05, Fig. 5A2), while EDP supplementation significantly reduced its abundance (P<0.01, Fig. 5A3). The relative abundance of Collinsella in group A was significantly higher than in groups D and E (P<0.05), with no significant differences among the other treatment groups (P>0.05), as shown in Fig. 5A4. Dietary protein levels did not significantly affect the abundance of Collinsella (P>0.05, Fig. 5A5), but EDP supplementation significantly reduced its relative abundance (P<0.01, Fig. 5A6).

#### Statistical analysis of KEGG metabolic pathways in cecal microbiota

**Fig. 6:**
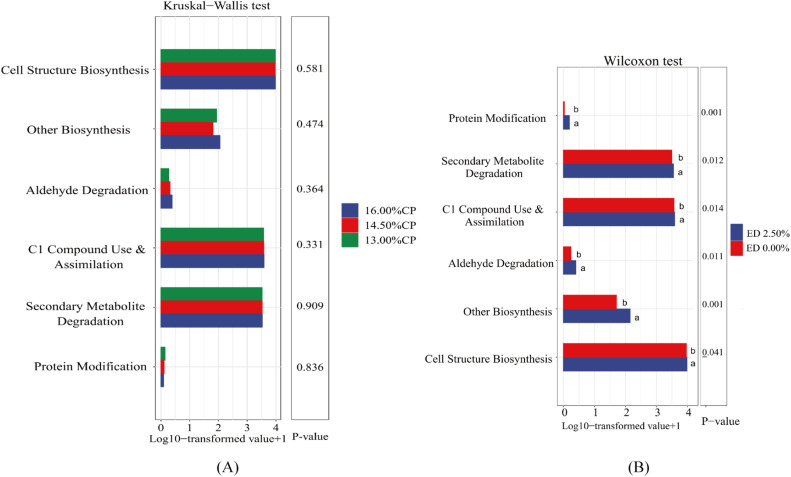
Statistical Graph of KEGG Metabolic Pathways in Cecal Microbiota. Fig. 6A displays the effect of Different Crude Protein Levels on Cecal Microbiota KEGG Metabolic Pathway Activities in Sanhua Geese. Fig. 6B illustrates effect of Dietary Edible Dock Powder Supplementation on Cecal Microbiota KEGG Metabolic Pathway Activities in Sanhua Geese.

Note: Different lowercase letters to the right of the same bar indicate significant differences (P < 0.05)

Fig. 6B shows the significant differences in the activity of cecal microbiota KEGG metabolic pathways due to dietary EDP supplementation. EDP supplementation significantly enhanced the activities of pathways related to protein modification, secondary metabolite degradation, C1 compound use and assimilation, aldehyde degradation, other biosynthesis, and cell structure biosynthesis (P < 0.05). Fig. 6A illustrates the activities of these pathways under different dietary CP levels. The dietary protein levels had no significant impact on the activities of cecal microbiota KEGG metabolic pathways in Sanhua geese (P > 0.05).

## Discussion

In the meat goose industry, low-protein diets are increasingly regarded as a resource-saving and cost-reducing strategy (Liang et al., 2023). However, low-protein diets may negatively affect the growth performance of geese, making it crucial to identify feed additives that can enhance their efficacy (Richman et al., 2015). In this context, EDP has emerged as a potential feed additive due to its high nutritional value and bioactive components. Our study demonstrated that EDP supplementation significantly improved the apparent digestibility of geese, particularly enhancing the apparent digestibility of gross energy (ADGE) in groups with lower protein levels (13 % crude protein, CP). Although there were no significant differences in the apparent digestibility of crude protein (ADCP) between the groups, the EDP-supplemented groups exhibited a trend of increased ADCP, likely due to the rich amino acids and nutrients in EDP promoting protein digestion and absorption. The improvement in energy digestibility directly contributed to enhanced feed efficiency (Ashour et al., 2020; Rolinec et al., 2018). This improvement in digestibility also had positive effects on the growth performance of geese. From weeks 5 to 8, as dietary protein levels decreased, ADFI and F/G showed significant reductions. Group A (16 % CP) had significantly higher ADFI and F/G compared to other groups. However, there were no significant differences in ADG among the groups, suggesting that the effects of low-protein diets on growth performance might not fully manifest during the early stages. From weeks 8 to 11, the benefits of EDP supplementation became more pronounced. ADG in groups D, E, and F was significantly higher than in group A, and F/G in group F was significantly lower than in groups A and C. These results indicate that EDP supplementation improved nutrient absorption and utilization, leading to increased body weight gain and feed efficiency. Over the entire 5 to 11-week period, group F exhibited significantly higher ADG than groups A, B, and C, with significantly lower F/G than groups A and C. Final body weight (FBW) in group F was also significantly higher than in group A. This further demonstrates the sustained positive effects of EDP supplementation on growth performance, likely mediated by improvements in apparent digestibility.

This study demonstrated that supplementing a low-protein diet with EDP does not negatively impact the carcass performance of Sanhua geese. On the contrary, it significantly improved carcass yield, eviscerated yield, and breast width in groups D and E. This enhancement is likely attributable to the rich bioactive compounds in EDP, such as polyphenols and flavonoids. These substances possess antioxidant and anti-inflammatory properties that enhance the digestibility and utilization of protein and energy in the feed, thus promoting bone growth and muscle deposition (Li et al., 2022). Additionally, the fermentation of EDP in the cecum of geese increases the content of polyphenols and flavonoids, further boosting their bioactivity (Li et al., 2023). The supplementation of EDP in a low-protein diet did not significantly affect the relative weights of the heart, spleen, gizzard, and intestines. However, it significantly reduced the relative liver weight of the geese. This reduction may be due to the regulatory effects of anthraquinones and antioxidant peptides present in EDP on liver metabolism. These bioactive substances can protect liver health and reduce oxidative stress and inflammatory responses through their antioxidant and anti-inflammatory mechanisms (Berillo et al., 2022).

The inclusion of EDP in low-protein diets has a significant impact on the serum biochemical parameters and breast muscle quality of Sanhua geese. The reduction in dietary protein levels notably decreased serum GLB levels and increased BUN levels. This finding is consistent with previous studies, suggesting that reduced protein intake may lead to an increased catabolism of endogenous proteins to meet metabolic needs, thereby elevating BUN concentrations (Saleh et al., 2021). The interaction between EDP and protein levels is complex, and the supplementation of EDP alone cannot fully mitigate the negative effects of a low-protein diet on GLB levels (Massarino et al., 2021).

In terms of breast muscle quality, lower dietary protein levels significantly reduced the pH value of the breast muscle. This reduction in pH is likely due to a decreased buffering capacity of the muscle as a result of reduced protein intake, leading to a more rapid postmortem decline in muscle pH (Ndazigaruye et al., 2019). EDP supplementation did not significantly affect the color parameters (L*, a*, b*) of the muscle, indicating that its effectiveness in improving meat color is limited.

The NMDS analysis revealed that the supplementation of EDP to low-protein diets significantly impacted the diversity and structure of the cecal microbiota in Sanhua geese. The groups without EDP supplementation exhibited similar microbial community structures, whereas the groups with EDP supplementation showed significantly different microbiota structures. This suggests that EDP may influence microbial community composition not only through direct antimicrobial effects but also by altering the metabolic environment of the gut. The results of the alpha diversity analysis showed that as dietary protein levels decreased, the richness and diversity of the cecal microbiota in geese significantly declined. This finding is consistent with previous studies, which indicate that high-protein diets provide more amino acids and nitrogen sources, supporting the growth of a diverse microbial community, thereby increasing gut microbiota diversity and richness (Wang et al., 2023). The supplementation of EDP significantly reduced the Chao1 and Shannon indices, indicating a negative impact on microbial diversity. This could be due to the polyphenolic compounds in EDP, which have antimicrobial properties and inhibit the growth of certain specific microorganisms (Cheng et al., 2023). Additionally, EDP contains a certain amount of antinutritional factors, which may alter the pH and microbial environment of the gut, further inhibiting microbial growth (Vasas et al., 2015).

This study found that supplementing low-protein diets with EDP significantly altered the cecal microbiota composition of geese, particularly affecting the abundance of Actinobacteria, *Collinsella*, and *Megamonas*. Actinobacteria maintain gut microbiota balance and inhibit the growth of harmful bacteria through the production of antibiotics. However, the anthraquinones in EDP, such as emodin and chrysophanol, have potent antibacterial properties that directly inhibit harmful bacteria, thereby reducing the host's reliance on Actinobacteria-produced antibiotics and leading to a decrease in their abundance (Ray and Mukherjee, 2021). *Collinsella* is usually associated with increased intestinal permeability and inflammation, but its abundance was significantly reduced under the influence of EDP. This is likely due to the anti-inflammatory effects of ellagic acid and quercetin in EDP, which reduce intestinal inflammation and thereby lower *Collinsella* abundance (Liang et al. 2010). *Megamonas*, an important fermenter of carbohydrates that produces short-chain fatty acids, also showed a significant decrease in abundance when dietary protein levels were reduced. This decrease might be due to the reduced availability of nitrogen and other nutrients in the gut, limiting the growth of *Megamonas* (Fusco et al. 2023). Additionally, EDP significantly reduced the abundance of *Megamonas*, potentially due to the sensitivity of these bacteria to the polyphenols and flavonoids in EDP, and the presence of antinutritional factors in EDP that alter the gut environment's pH and available metabolites, reducing the conditions favorable for their growth (Plamada and Vodnar. 2021).

This study demonstrates that the supplementation of EDP in the diet significantly impacts the activity of KEGG metabolic pathways in the cecal microbiota of Sanhua geese, particularly enhancing the pathways related to protein modification, secondary metabolite degradation, C1 compound utilization and assimilation, aldehyde degradation, other biosynthesis, and cell structure biosynthesis. The enhancement of these pathways suggests that the microbiota plays a more active role in regulating protein function and metabolism, reducing the accumulation of harmful substances, improving metabolic flexibility and energy efficiency, protecting intestinal barrier function, and maintaining nutritional balance and metabolic needs of the host (Hu et al., 2024; Liang and Kitts, 2014; Bang and Lee, 2018).

Despite the significant enhancement of these critical metabolic pathways by EDP, this study found that different dietary crude protein levels did not significantly affect the KEGG metabolic pathway activity in the cecal microbiota. A previous study also reported that changes in protein levels have minimal direct impact on the metabolic pathways of gut microbiota, possibly because gut microbiota has strong adaptability and can maintain its functional stability by adjusting its metabolic pathways in response to nutritional changes (He et al., 2023).

## Conclusion

In conclusion, EDP supplementation in low-protein diets significantly improves growth performance, digestibility, and microbiota composition in Sanhua geese. These findings support the potential of EDP as a valuable feed additive, offering a sustainable and cost-effective solution for enhancing livestock productivity. Future research should focus on optimizing the use of EDP in various livestock species and rearing conditions to fully harness its benefits.

## Declaration of competing interest

All authors have read and approved the manuscript, and there are no conflicts of interest to declare.
